# Treatment effect of palbociclib plus endocrine therapy by prognostic and intrinsic subtype and biomarker analysis in patients with bone-only disease: a joint analysis of PALOMA-2 and PALOMA-3 clinical trials

**DOI:** 10.1007/s10549-020-05782-4

**Published:** 2020-08-11

**Authors:** Richard S. Finn, Massimo Cristofanilli, Johannes Ettl, Karen A. Gelmon, Marco Colleoni, Carla Giorgetti, Eric Gauthier, Yuan Liu, Dongrui R. Lu, Zhe Zhang, Cynthia Huang Bartlett, Dennis J. Slamon, Nicholas C. Turner, Hope S. Rugo

**Affiliations:** 1 Division of Hematology/Oncology, David Geffen School of Medicine at UCLA, 2825 Santa Monica Blvd, Suite 200, Santa Monica, CA USA; 2 Robert H. Lurie Comprehensive Cancer Center, Feinberg School of Medicine, 710 N Fairbanks Ct, Suite 8-250A, Chicago, IL USA; 3 Department of Obstetrics and Gynecology, School of Medicine, Klinikum Rechts der Isar, Technical University of Munich, Ismaninger Str 22, 81675 Munich, Germany; 4 British Columbia Cancer Agency, 675 West 10th Ave, Vancouver, BC Canada; 5 IEO European Institute of Oncology, IRCCS, Via Ripamonti 435, Milan, Italy; 6 Pfizer Italia, Via Anna Maria Mozzoni, 12, Milan, Italy; 7 Pfizer Inc, 525 Market Street, San Francisco, CA USA; 8 Pfizer Inc, 10646 Science Center Dr, La Jolla, San Diego, CA USA; 9 Pfizer Inc, 500 Arcola Rd, Collegeville, PA USA; 10 Royal Marsden Hospital and Institute of Cancer Research, Fulham Rd, London, SW3 6JJ UK; 11 University of California San Francisco Comprehensive Center, 1600 Divisadero St, San Francisco, CA USA

**Keywords:** Bone-only disease, Disease-free interval, Intrinsic subtype, Palbociclib, Treatment-free interval

## Abstract

**Purpose:**

This analysis evaluated the relationship between treatment-free interval (TFI, in PALOMA-2)/disease-free interval (DFI, in PALOMA-3) and progression-free survival (PFS) and overall survival (OS, in PALOMA-3), treatment effect in patients with bone-only disease, and whether intrinsic subtype affects PFS in patients receiving palbociclib.

**Methods:**

Data were from phase 3, randomized PALOMA-2 and PALOMA-3 clinical studies of hormone receptor‒positive/human epidermal growth factor receptor 2‒negative (HR+ /HER2−) advanced breast cancer (ABC) patients receiving endocrine therapy plus palbociclib or placebo. Subpopulation treatment effect pattern plot (STEPP) analysis evaluated the association between DFI and PFS and OS. PFS by luminal subtype and cyclin-dependent kinase (CDK) 4/6 or endocrine pathway gene expression levels were evaluated in patients with bone-only disease; median PFS and OS were estimated by the Kaplan–Meier method.

**Results:**

Median durations of TFI were 37.1 and 30.9 months (PALOMA-2) and DFI were 49.2 and 52.0 months (PALOMA-3) in the palbociclib and placebo groups, respectively. Among the PALOMA-2 biomarker population (*n* = 454), 23% had bone-only disease; median PFS was longer with palbociclib versus placebo (31.3 vs 11.2 months; hazard ratio, 0.41; 95% CI 0.25‒0.69). The interaction effect of bone-only versus visceral disease subgroups on median PFS with palbociclib was not significant (*P* = 0.262). Among the PALOMA-3 biomarker population (*n* = 302), 27% had bone-only disease. STEPP analyses showed that palbociclib PFS benefit was not affected by DFI, and that palbociclib OS effect may be smaller in patients with short DFIs. Among patients who provided metastatic tumor tissues (*n* = 142), regardless of luminal A (hazard ratio, 0.23; 95% CI 0.11‒0.47; *P* = 0.0000158) or luminal B (hazard ratio, 0.26; 95% CI 0.12‒0.56; *P* = 0.000269) subtype, palbociclib improved PFS versus placebo.

**Conclusions:**

These findings support palbociclib plus endocrine therapy as standard of care for HR+ /HER2− ABC patients, regardless of baseline TFI/DFI or intrinsic molecular subtype, including patients with bone-only disease.

**Trial registration:**

Pfizer (clinicaltrials.gov:NCT01740427, NCT01942135).

## Background

Selecting the optimal treatment approach in patients with advanced breast cancer (ABC) is challenging because of the lack of valid predictors of long-term survival [1]. Many patients with hormone receptor‒positive/human epidermal growth factor receptor 2‒negative (HR+ /HER2−) ABC respond to endocrine therapy (ET), a mainstay treatment for these cancers [2, 3]. Both biomarkers and clinical parameters have been evaluated as potential predictors of ET benefit [4]. Estrogen and progesterone receptor and HER2 status are still the most important biomarkers predictive of benefit from ET [5–7]. More recently, various intrinsic molecular subtypes of breast cancer have been characterized (luminal A, luminal B, HER2-enriched, basal-like, normal-like, among others) to assess potential differences in patient prognosis and treatment response [8, 9]. Luminal molecular subtype tumors comprise the majority of breast cancers (83%) [10] and are associated with substantially better outcomes than other molecular subtypes [8, 11]. Additionally, luminal tumors are associated with longer disease-free intervals (DFIs), a predictor of better prognosis in patients with de novo and recurrent ABC [11, 12].

With regard to clinical parameters for patients with HR+ ABC, bone-only metastases are usually associated with a prolonged natural history compared with those with visceral disease [13], and these patients are frequently considered candidates for single-agent ET [14]. Patients with HR+/HER2− ABC presenting with visceral metastases typically have a worse prognosis than patients without visceral metastases [15] and are often initially treated with chemotherapy in spite of their hormonal receptor status [14] and regardless of guidelines suggesting the importance of endocrine-based therapy for this group of patients [3]. In addition, patients with de novo metastatic disease or patients with a longer DFI have a better prognosis than those with a shorter DFI or recurrent metastatic breast cancer [16, 17]. Nevertheless, many patients receiving systemic therapies, including ET or chemotherapy, relapse or eventually develop resistance [18, 19].

Palbociclib is a first-in-class cyclin-dependent kinase (CDK) 4/6 inhibitor that blocks cell cycle progression from G1 to S phase and has shown synergistic activity with antiestrogens [20, 21]. In the PALOMA-2 and PALOMA-3 clinical trials, palbociclib plus ET significantly improved progression-free survival (PFS) versus ET alone in patients with HR+ /HER2− ABC [22, 23]. In PALOMA-2, median PFS was 27.6 months with palbociclib plus letrozole as first-line ABC therapy versus 14.5 months with placebo plus letrozole (hazard ratio, 0.56; *P* < 0.0001; data cutoff, May 31, 2017) [24]. In PALOMA-3, median PFS was 11.2 months with palbociclib plus fulvestrant versus 4.6 months with placebo plus fulvestrant in patients with ABC whose disease had progressed following ET (hazard ratio, 0.50; *P* < 0.0001; data cutoff, October 23, 2015) [25]. Additionally, median overall survival (OS) was 34.9 months with palbociclib plus fulvestrant compared with 28.0 months with placebo plus fulvestrant (hazard ratio, 0.81; *P* = 0.09; data cutoff, April 13, 2018) [26].

Identifying subgroups of patients and intrinsic molecular subtypes of breast cancer that are sensitive to or resistant to palbociclib treatment is important to optimize patients’ therapy selection. It is also important to characterize subgroups of patients who benefit from ET alone and might not require combination treatment options. This analysis evaluated the relationship between initial treatment-free interval (TFI, in PALOMA-2) or DFI (in PALOMA-3) and PFS and OS outcomes (in PALOMA-3). A biomarker analysis of CDK4/6 and endocrine pathways was performed to examine palbociclib treatment effect in patients with bone-only versus visceral disease. Additionally, the effect of luminal subtypes of breast cancer on palbociclib treatment benefit was assessed.

## Methods

### Study design and patients

This analysis included data from the phase 3, double-blind, placebo-controlled, randomized PALOMA-2 (NCT01740427) and PALOMA-3 (NCT01942135) clinical studies [22, 23]. PALOMA-2 included postmenopausal women (*n* = 666) with previously untreated estrogen receptor‒positive/HER2‒ ABC who were randomized 2:1 to receive palbociclib (125 mg/day, 3 weeks on/1 week off schedule) plus letrozole (2.5 mg/day) or placebo plus letrozole [23]. In PALOMA-3, women (*n* = 521) of any menopausal status with HR+/HER2− ABC whose disease had progressed after previous ET were randomized 2:1 to receive palbociclib (125 mg/day, 3/1 schedule) plus fulvestrant (500 mg on days 1 and 15 of cycle 1 and on day 1 of each subsequent cycle) or placebo plus fulvestrant [22]. Microarray data from the PALOMA-2 and PALOMA-3 studies were previously deposited in Gene Expression Omnibus (accession numbers GSE133394 and GSE128500, respectively); 568 baseline tumor tissues from either primary or metastatic biopsies were collected from patients in PALOMA-2 [27] and 462 tumor samples were collected, including 302 evaluable samples (53% archival primary samples and 47% metastatic biopsy samples), from PALOMA-3 [28].

In the PALOMA-2 study, visceral metastases referred to any lung (including pleura) or liver involvement. Bone-only disease was defined as bone lesions confirmed by computed tomography (CT), magnetic resonance imaging (MRI), or bone X-ray, and nonvisceral disease was defined as the absence of lung (including pleura) or liver involvement with the exclusion of any patient with bone-only disease. In the PALOMA-3 study, visceral metastases referred to lung, liver, brain, pleural, and peritoneal involvement. Bone-only disease was defined as lytic or mixed lytic-blastic lesions that could be accurately assessed by CT or MRI, and nonvisceral disease was defined as absence of lung, liver, brain, pleural, or peritoneal involvement with the exclusion of any patient with bone-only disease.

### Analysis populations

The intent-to-treat (ITT) population included all patients who were randomized, with study treatment assignment designated according to initial randomization. The biomarker population in PALOMA-2 was defined as a subset of the safety population who had a baseline value for ≥ 1 biomarker. In PALOMA-3, the biomarker population comprised a subset of the safety population who had both baseline and ≥ 1 follow-up value for ≥ 1 biomarker. An analysis of PFS by luminal subtype was also performed among patients from PALOMA-3 who had provided metastatic disease tumor tissues. The TFI analysis was performed in patients who received adjuvant therapy in PALOMA-2 and the DFI and subpopulation treatment effect pattern plot (STEPP) analyses were performed in patients who received adjuvant therapy in PALOMA-3. STEPP analyses of TFI data have been previously published [24].

### Treatment-free interval/disease-free interval analysis

**Fig. 1:**
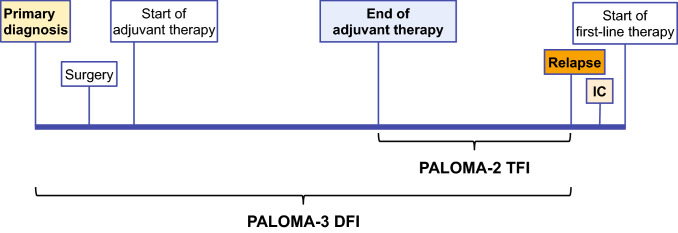
TFI and DFI definitions. *DFI* disease-free interval, *IC* informed consent, *TFI* treatment-free interval

Treatment-free interval was defined as the time between the end of any (neo)adjuvant therapy and relapse, and DFI was defined as the time between the first diagnosis of breast cancer and disease recurrence (Fig. 1). An analysis based on DFI versus TFI was performed for PALOMA-3 to correspond with the baseline DFI patient characteristics presented in the initial PALOMA-3 primary manuscript [29].

The exploratory STEPP [30] is a statistical method to explore treatment by covariate interactions from two treatment arms. The method is based on constructing overlapping subpopulations of patients with respect to a covariate of interest and observing the pattern of the treatment effects estimated across subpopulations. STEPP analyses were conducted in the ITT population using data from patients who received (neo)adjuvant therapy to evaluate the effect of DFI in PALOMA-3 assessed as continuous variables on PFS and OS outcomes. STEPP analyses evaluating PFS effect in patients with visceral versus nonvisceral (excluding bone-only) metastases were also conducted.

### Luminal subtype analyses

In PALOMA-2, patients provided freshly biopsied formalin-fixed paraffin-embedded (FFPE) tissue from metastatic or recurrent tumor lesions whenever possible; however if a tissue sample was unavailable, the study investigator could recommend a de novo fresh biopsy [27, 31]. In PALOMA-3, all patients provided FFPE tissue from metastatic disease, except for patients with bone-only disease or relapse while on adjuvant therapy and who had surgery within 3 years who could provide archival primary tissue [28].

Luminal subtypes were determined by gene expression profiles from FFPE tumor tissue [27, 28, 31]. The EdgeSeq Oncology Biomarker Panel (HTG Molecular Diagnostics; Tucson, AZ, USA) was used for gene expression profiling [27, 28]. The intrinsic molecular subtypes were determined using the absolute intrinsic molecular subtyping algorithm through a set of binary rules that compared expression measurements for pairs of genes from each individual patient [27, 28, 31, 32].

The Kaplan–Meier method was used to estimate median PFS by luminal subtype. To assess the effect of luminal subtype in metastatic disease, PFS by luminal subtype only among patients who provided metastatic disease tumor tissues was evaluated in PALOMA-3. Intrinsic subtype analysis using data from PALOMA-2 have been previously reported [27]. Hazard ratios and 95% confidence intervals (CIs) were computed using the Brookmeyer and Crowley method.

### Analyses by bone-only, nonvisceral (excluding bone-only), and visceral disease

Median PFS for each treatment arm was estimated and biomarker analyses of CDK4/6 and endocrine pathways were compared in patients with bone-only disease, nonvisceral disease (excluding bone-only), or visceral disease. Cox proportional hazards models were performed to evaluate the interaction between disease site at baseline (bone only, visceral, or nonvisceral that excluded bone only) and treatment effect, as well as the interaction between prespecified baseline gene expression (eg, cyclin-dependent kinase 4 [*CDK4*], estrogen receptor 1 [*ESR1*]*,* cyclin-dependent kinase 6 [*CDK6*]*,* cyclin D1 [*CCND1*]*,* cyclin D3 [*CCND3*]*,* cyclin E1 [*CCNE1*]) and treatment effect in patients with bone-only, nonvisceral, and visceral disease, respectively; in the biomarker population, the model included interaction terms of treatment by gene expression with main effect terms “treatment” and “gene expression.” Gene expression data were quantile normalized and log2 transformed. Interaction *P* values were reported. No adjustments for multiple comparisons were made because of the exploratory nature of the analyses. Statistical tests were two-sided with *P* < 0.05 considered significant.

## Results

### PALOMA-2

In total, 418 patients (62.8%) received adjuvant therapy and were included in the TFI analysis (data cutoff, May 31, 2017; Table 1) and 454 patients were included in the biomarker population. The median duration of TFI was 37.1 months in the palbociclib plus letrozole group and 30.9 months in the placebo plus letrozole group. Overall, 35.4% and 34.0% of patients in the palbociclib and placebo groups, respectively, had a TFI of ≤ 1 year and 32.5% and 32.6% had a TFI of > 5 years, respectively. Previously published STEPP analyses indicated that TFI did not affect palbociclib treatment outcomes (PFS) in the overall population or in patients with visceral or nonvisceral metastases who received adjuvant therapy [24].

**Table 1:** Summary of baseline TFI/DFI

PALOMA-2	Palbociclib + letrozole (*n* = 277)	Placebo + letrozole (*n* = 141)
TFI, *n* (%)		
≤ 1 year	98 (35.4)	48 (34.0)
> 1‒2 years	25 (9.0)	16 (11.3)
> 2‒5 years	64 (23.1)	31 (22.0)
> 5 years	90 (32.5)	46 (32.6)
Duration of TFI, months		
Mean (SD)	50.2 (59.9)	55.6 (69.9)
Median (range)	37.1 (− 10.7 to 337.5)	30.9 (− 3.6 to 332.7)

PALOMA-3	Palbociclib + fulvestrant (*n* = 232)	Placebo + fulvestrant (*n* = 123)
DFI, *n* (%)		
≤ 1 year	10 (4.3)	3 (2.4)
> 1‒2 years	30 (12.9)	19 (15.4)
> 2‒5 years	101 (43.5)	49 (39.8)
> 5‒10 years	53 (22.8)	34 (27.6)
> 10 years	37 (15.9)	18 (14.6)
Duration of DFI, months		
Mean (SD)	65.9 (54.0)	69.6 (57.4)
Median (range)	49.2 (0.03‒277.9)	52.0 (2.8‒326.4)

TFI was defined as the time between the end of any (neo)adjuvant therapy and relapse in PALOMA-2, and DFI was defined as the time between the first diagnosis of breast cancer and disease recurrence in PALOMA-3

*DFI* disease-free interval, *TFI* treatment-free interval

**Fig. 2:**
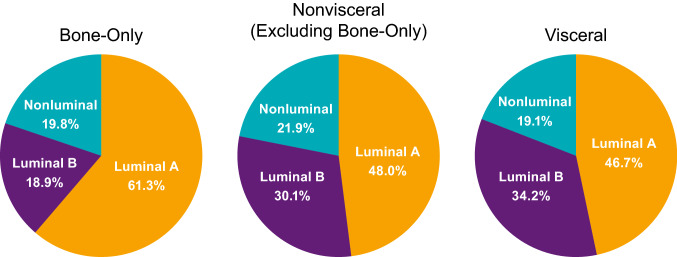
PALOMA-2: breast cancer subtype distribution by bone-only, nonvisceral (excluding bone-only), and visceral disease^a^. ^a^Biomarker population; *n* = 454

Of the 454 patients included in the biomarker population, 303 received palbociclib plus letrozole and 151 received placebo plus letrozole. Most patients with bone-only disease in the biomarker population had luminal A tumors (61.3%), 18.9% had luminal B tumors, and 19.8% had nonluminal tumors (Fig. 2). Luminal A disease was also more common than luminal B and nonluminal disease in patients with visceral disease.

In the ITT population, bone-only disease was reported in 23% (*n* = 150) of patients. Median PFS was prolonged with palbociclib plus letrozole compared with placebo plus letrozole in patients with bone-only, visceral disease, and nonvisceral (excluding bone-only) disease, respectively, but the interaction effect was not statistically significant (*P* = 0.240; Table 2). In the biomarker population, the improvement in median PFS with the addition of palbociclib to letrozole appeared to be more profound in patients with bone-only disease compared with those with visceral disease or nonvisceral disease, but the interaction effect was not statistically significant (*P* = 0.262).

**Table 2:** Median PFS in patients with bone-only, nonvisceral (excluding bone-only), and visceral disease

	PALOMA-2			PALOMA-3		
	Palbociclib + letrozole	Placebo + letrozole	Interaction *P* value	Palbociclib + fulvestrant	Placebo + fulvestrant	Interaction *P* value
ITT population						
Bone-only, *n*	102	48	0.240	86	38	0.471
Median PFS (95% CI), months	36.2 (27.6–NE)	11.2 (8.2–22.0)		14.3 (11.2–NE)	9.2 (4.8–20.0)	
Hazard ratio (95% CI)	0.40 (0.26–0.62)	0.40 (0.26–0.62)		0.64 (0.38–1.06)	0.64 (0.38–1.06)	
Nonvisceral (excluding bone-only), *n*	128	64		61	32	
Median PFS (95% CI), months	33.4 (27.6–NE)	23.5 (13.8–30.6)		16.6 (11.1–NE)	5.6 (3.5–9.3)	
Hazard ratio (95% CI)	0.60 (0.40–0.89)	0.60 (0.40–0.89)		0.40 (0.23–0.73)	0.40 (0.23–0.73)	
Visceral, *n*	214	110		200	104	
Median PFS (95% CI), months	19.3 (16.4–24.2)	12.3 (8.4–16.4)		9.2 (7.5–11.1)	3.4 (1.9–5.1)	
Hazard ratio (95% CI)	0.61 (0.46–0.80)	0.61 (0.46–0.80)		0.47 (0.36–0.62)	0.47 (0.36–0.62)	
Biomarker population						
Bone-only, n	72	34	0.262	54	27	0.363
Median PFS (95% CI), months	31.3 (23.9–NE)	11.2 (5.5–22.0)		16.6 (11.2–NE)	11.2 (4.8–20.0)	
Hazard ratio (95% CI)	0.41 (0.25–0.69)	0.41 (0.25–0.69)		0.79 (0.41–1.60)	0.79 (0.41–1.60)	
Nonvisceral (excluding bone-only), *n*	83	40		32	21	
Median PFS (95% CI), months	27.7 (21.9–NE)	21.9 (13.8–30.6)		NE (9.1–NE)	5.5 (1.9–NE)	
Hazard ratio (95% CI)	0.63 (0.39–1.03)	0.63 (0.39–1.03)		0.44 (0.20–0.96)	0.44 (0.20–0.96)	
Visceral, *n*	148	77		108	60	
Median PFS (95% CI), months	19.2 (14.0–24.2)	11.3 (8.3–16.6)		9.5 (7.5–12.1)	2.2 (1.9–4.2)	
Hazard ratio (95% CI)	0.67 (0.49–0.93)	0.67 (0.49–0.93)		0.46 (0.32–0.67)	0.46 (0.32–0.67)	

*CI* confidence interval, *ITT* intent-to-treat, *NE* not estimable,* PFS* progression-free survival

In the biomarker population, mRNA levels at baseline of *CDK4*, *ESR1*, *CDK6*, *CCND1*, *CCND3*, and *CCNE1* were similar between patients with bone-only disease and visceral disease (Table 3). In patients with visceral disease, *CDK4* gene expression was associated with letrozole resistance (*P* = 0.010; Table 3). No statistically significant treatment interactions were observed for any other gene in any of the patient subgroups.

**Table 3:** Gene expression levels at baseline and treatment effect interaction

	PALOMA-2			PALOMA-3		
	Bone-only (*n* = 106)	Nonvisceral (excluding bone-only) (*n* = 123)	Visceral (*n* = 225)	Bone-only (*n* = 81)	Nonvisceral (excluding bone-only) (*n* = 53)	Visceral (*n* = 168)
Baseline gene mRNA expression levels, median (minimum, maximum)						
*CDK4*	10.3 (8.6, 11.2)	10.2 (8.5, 11.4)	10.4 (8.3, 11.9)	11.3 (9.4, 14.2)	11.4 (10.1, 12.9)	11.3 (9.7, 13.2)
*ESR1*	12.7 (8.6, 16.0)	13.4 (8.7, 16.0)	13.1 (7.5, 16.7)	13.2 (8.8, 16.5)	12.8 (9.3, 17.3)	13.4 (8.6, 16.5)
*CDK6*	8.7 (6.3, 10.6)	8.7 (4.2, 10.6)	8.7 (3.9, 10.5)	9.9 (8.7, 12.7)	9.9 (8.8, 12.2)	9.9 (8.1, 12.1)
*CCND1*	12.4 (8.8, 16.7)	12.7 (8.5, 16.7)	12.6 (8.6, 16.7)	13.0 (9.1, 16.5)	12.9 (8.4, 17.3)	12.9 (9.0, 17.3)
*CCND3*	10.1 (8.2, 11.6)	10.1 (8.3, 12.2)	10.1 (8.2, 11.6)	10.6 (8.1, 12.5)	10.4 (9.0, 12.0)	10.3 (8.6, 12.2)
*CCNE1*	6.7 (3.7, 9.3)	6.8 (2.2, 8.7)	7.0 (3.2, 11.5)	6.6 (1.9, 9.6)	7.2 (4.1, 9.7)	6.9 (1.9, 9.9)
Treatment interaction, *P* value						
*CDK4*	0.601	0.518	0.010	0.161	0.223	0.735
*ESR1*	0.745	0.079	0.643	0.468	0.530	0.287
*CDK6*	0.718	0.526	0.338	0.008	0.818	0.654
*CCND1*	0.493	0.562	0.823	0.479	0.555	0.311
*CCND3*	0.946	0.153	0.626	0.101	0.341	0.239
*CCNE1*	0.891	0.578	0.196	0.114	0.036	0.004

*CCND1* cyclin D1, *CCND3* cyclin D3, *CCNE1* cyclin E1, *CDK4* cyclin-dependent kinase 4, *CDK6* cyclin-dependent kinase 6, *ESR1* estrogen receptor 1

### PALOMA-3

**Fig. 3:**
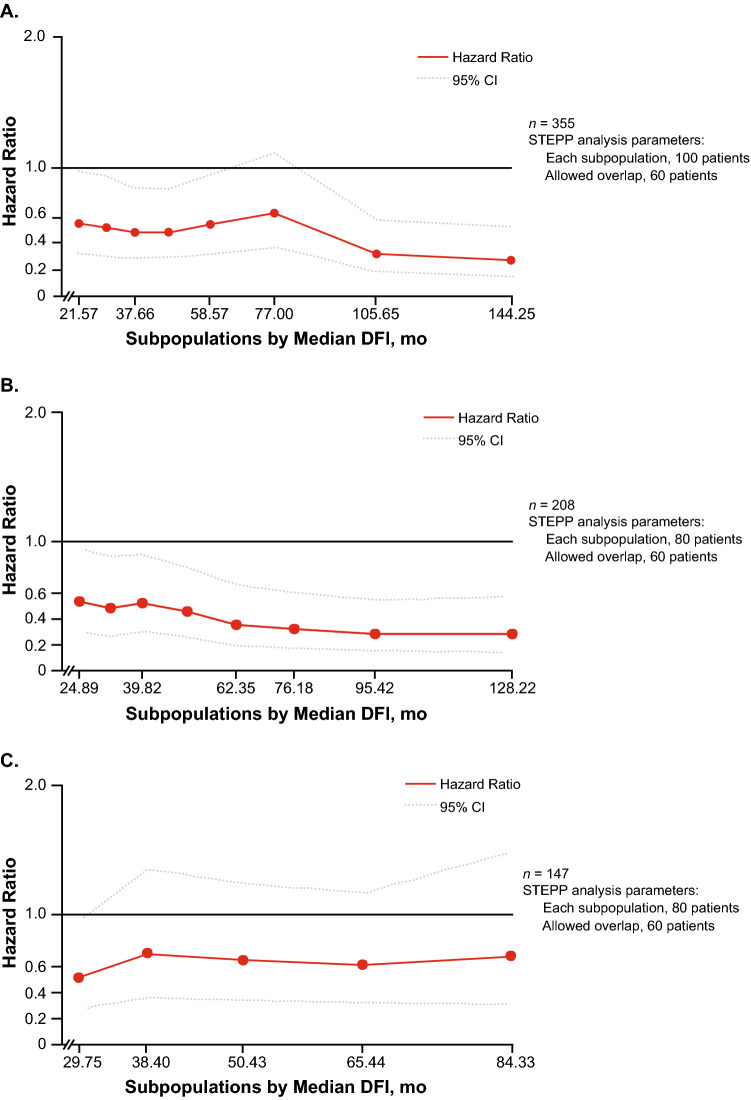
PALOMA-3: STEPP Analyses Evaluating PFS Effect. **a** Patients who received adjuvant therapy, **b** patients who received adjuvant therapy and had visceral metastases, and **c** patients who received adjuvant therapy and had nonvisceral metastases^a^. *CI* confidence interval, *DFI* disease-free interval, *PFS* progression-free survival, *STEPP* subpopulation treatment effect pattern plot. ^a^DFI was defined as the time between the first diagnosis of breast cancer and disease recurrence in PALOMA-3

**Fig. 4:**
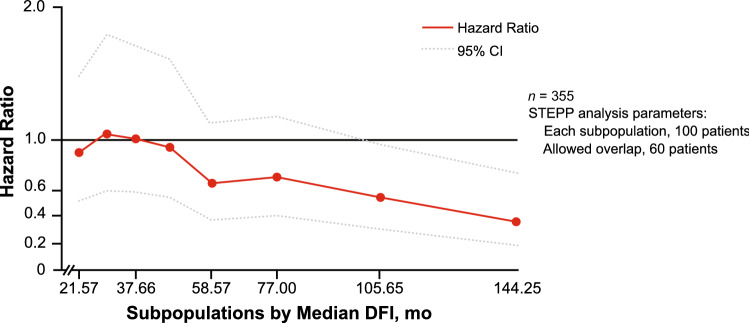
PALOMA-3: STEPP analyses evaluating OS effect in patients who received adjuvant therapy^a^. *CI* confidence interval, *DFI* disease-free interval, *OS* overall survival, *STEPP* subpopulation treatment effect pattern plot. ^a^DFI was defined as the time between the first diagnosis of breast cancer and disease recurrence in PALOMA-3

A total of 355 patients (68.1%) received previous (neo)adjuvant therapy and were included in the DFI analysis (data cutoff, October 23, 2015 for PFS and May 24, 2018 for OS; Table 1) and a total of 302 patients were included in the biomarker population. The median duration of DFI was similar between the palbociclib plus fulvestrant group (49.2 months) and the placebo plus fulvestrant group (52.0 months). Most patients in either treatment arm had a DFI of > 2 years. STEPP analyses of the PFS treatment effect of palbociclib indicated that PFS benefit was not affected by DFI in all patients who had received adjuvant therapy, regardless of whether they had visceral or nonvisceral disease (Fig. 3). In contrast, STEPP analyses of the OS treatment effect of palbociclib suggested that the OS benefit may be smaller in patients with short DFIs (Fig. 4).

**Fig. 5:**
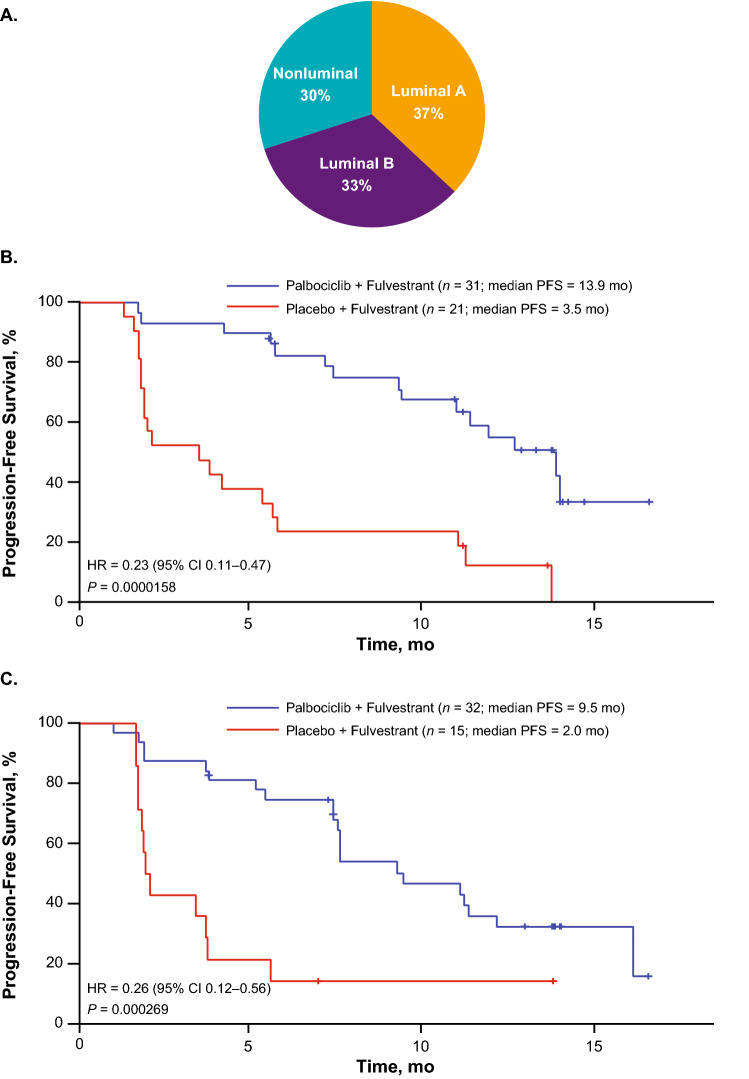
PALOMA-3: PFS by Subtype Distribution. **a** Intrinsic subtype distribution of tumors and PFS by **b** luminal A or **c** luminal B subtype among patients who provided metastatic disease tumor tissues (*n* = 142). *CI* confidence interval, *HR* hazard ratio, *PFS* progression-free survival

Of the 462 tumor samples analyzed (from 521 patients), 142 (47%) were metastatic biopsy samples. Among the patients who provided metastatic disease tumor tissues, 37% were luminal A subtype, 33% were luminal B subtype, and 30% had nonluminal tumor type (Fig. 5a). Among patients who provided metastatic disease tumor tissues, palbociclib plus fulvestrant improved PFS compared with placebo plus fulvestrant, regardless of luminal A or luminal B tumor subtype (Fig. 5b, c). In patients with luminal A tumors, the median PFS was 13.9 months in the palbociclib plus fulvestrant group versus 3.5 months in the placebo plus fulvestrant group (hazard ratio, 0.23; 95% CI 0.11‒0.47; *P* = 0.0000158). Among patients with luminal B tumors, the median PFS was 9.5 months in the palbociclib plus fulvestrant group versus 2.0 months in the placebo plus fulvestrant group (hazard ratio, 0.26; 95% CI 0.12‒0.56; *P* = 0.000269).

**Fig. 6:**
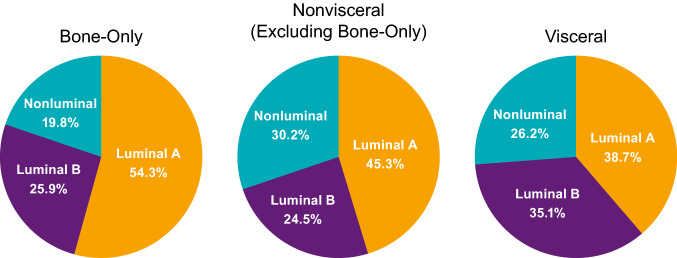
PALOMA-3: breast cancer subtype distribution by bone-only, nonvisceral (excluding bone-only), and visceral disease (*n* = 302)

Of the 302 patients included in the biomarker population, 194 patients were included in the palbociclib plus fulvestrant group and 108 in the placebo plus fulvestrant group. Luminal A subtype was more prominent in patients with bone-only disease (54.3% vs 25.9%) and similar in those with visceral disease compared with patients with luminal B subtype (38.7% vs 35.1%; Fig. 6). In patients with bone-only disease, 19.8% had nonluminal tumor subtype; in those with visceral disease, 26.2% had nonluminal tumor types.

Bone-only disease was reported in 24% (*n* = 124) of patients in the ITT population. Median PFS was longer in the palbociclib plus fulvestrant group compared with the placebo plus fulvestrant group in patients with bone-only, visceral disease, and nonvisceral (excluding bone-only), respectively (Table 2). In the biomarker population, 27% (*n* = 81) of patients had bone-only disease. Similar to findings in the ITT population, median PFS was longer with palbociclib plus fulvestrant compared with placebo plus fulvestrant in both the bone-only and visceral subgroups. Median PFS was not reached in the palbociclib plus fulvestrant group in patients with nonvisceral (excluding bone-only) disease. Among patients with bone-only disease, the median PFS was 16.6 months in the palbociclib plus fulvestrant group compared with 11.2 months in the placebo plus fulvestrant group (hazard ratio, 0.79; 95% CI 0.41‒1.60; Table 2).

Baseline mRNA levels of *CDK4*, *ESR1*, *CDK6, CCND1 CCND3,* and *CCNE1* were similar between patients with bone-only, nonvisceral (excluding bone-only), and visceral disease in the biomarker population (Table 3). Statistically significant treatment interactions were observed for *CDK6* in the bone-only disease subgroup and for *CCNE1* in the visceral subgroup (Table 3).

## Discussion

The introduction of CDK4/6 inhibitors for the treatment of HR+/HER2− ABC has made a substantial impact on patients’ outcomes [23, 33–35]. The current analyses suggest that palbociclib plus ET improved PFS compared with ET plus placebo in patients who received adjuvant therapy and developed disease recurrence as well as in patients with bone-only disease and visceral disease. The degree of benefit observed was consistent across different lengths of TFI (PALOMA-2) or DFI (PALOMA-3), regardless of whether patients had visceral or nonvisceral metastases [24].

Previous findings have shown that patients with luminal subtype disease benefit from the combination of palbociclib plus letrozole, regardless of luminal A or luminal B subtype [27, 31]. In patients with luminal A tumors, the median PFS was 30.4 months with palbociclib plus letrozole compared with 17.0 months with placebo plus letrozole (hazard ratio, 0.55; 95% CI 0.39‒0.77; *P* = 0.000547) [27, 31]. Patients with luminal B tumors had a median PFS of 19.6 months in the palbociclib plus letrozole group compared with 11.0 months in the placebo plus letrozole group (hazard ratio, 0.51; 95% CI 0.34‒0.77; *P* = 0.00109) [27, 31]. Additionally, a previous analysis of patients from the PALOMA-3 study who provided primary or metastatic samples demonstrated that patients with luminal A or luminal B subtypes both benefited from palbociclib plus fulvestrant (luminal A: median PFS was 16.6 months with palbociclib plus fulvestrant versus 4.8 months with placebo plus fulvestrant [hazard ratio, 0.41; 95% CI 0.25‒0.66]; luminal B: median PFS was 9.2 months with palbociclib plus fulvestrant versus 3.5 months with placebo plus fulvestrant [hazard ratio, 0.64; 95% CI 0.38‒1.09]) [28]. Together with present results by luminal subtype in patients who provided metastatic disease tumor tissues and received palbociclib plus fulvestrant, these findings highlight that, despite clear differences in prognosis, the magnitude of palbociclib plus ET effect was similar in luminal A and luminal B tumor subtypes.

The present analysis adds to the current body of literature evaluating clinical subgroups or tumor biomarkers that may identify patients who benefit from palbociclib combination treatment. Patients with ABC who present with visceral metastases generally have a worse prognosis than patients without visceral metastases [15]. However, consistent with the current findings, previous subgroup analyses of patients with and without visceral metastases or with bone-only disease showed significant improvements in median PFS with palbociclib plus ET compared with placebo plus ET [24, 36]. In a previous biomarker analysis of tumor tissues from PALOMA-2, no predictive biomarker was associated with lack of benefit from palbociclib plus letrozole treatment, but higher *CDK4* expression was identified as a marker of resistance to treatment with letrozole alone [27, 31]. A similar biomarker analysis of patients in PALOMA-3 showed that palbociclib plus fulvestrant was effective in all biomarker groups assessed, but high *CCNE1* mRNA expression was associated with relative resistance to palbociclib plus fulvestrant [28].

Longer DFIs are associated with significantly longer survival rates in patients with breast cancer [16]. STEPP analyses from both the PALOMA-2 and PALOMA-3 studies suggested that the PFS treatment effect of palbociclib was not affected by the length of TFI or DFI in all patients who had received adjuvant therapy, regardless of whether they had visceral or nonvisceral metastases [24]. Similarly, findings from MONALEESA-2, a phase 3, double-blind, randomized study of postmenopausal women with HR+/HER2− ABC, demonstrated that the PFS benefit associated with ribociclib plus letrozole compared with placebo plus letrozole was consistent in patients with a TFI of ≤ 24 months versus > 24 months, ≤ 36 months versus > 36 months, and ≤ 48 months versus > 48 months [37]. In contrast, the MONARCH-3 study, a phase 3, double-blind, randomized study of patients with HR+/HER2− ABC, showed that PFS benefit for abemaciclib plus a nonsteroidal aromatase inhibitor versus placebo plus a nonsteroidal aromatase inhibitor was lower in subgroups of patients with a baseline TFI < 36 months compared with ≥ 36 months [38]. However, the MONARCH-3 study had a shorter median duration of follow-up (17.8 months) [38] than the PALOMA-2 and PALOMA-3 analyses. In contrast to the PFS treatment effect results, the STEPP analyses from PALOMA-3 suggested that the OS treatment effect of palbociclib may be smaller in patients with short DFIs. These findings are consistent with previously published OS results from PALOMA-3 showing that patients with a DFI of > 24 months derived greater benefit from palbociclib plus fulvestrant than patients with a DFI of ≤ 24 months [26].

Regardless of whether patients had luminal A or luminal B subtype tumors, palbociclib plus ET improved PFS compared with placebo plus ET [27, 31]. HR+ status is the common driver in luminal disease [10]. These data support the investigation of palbociclib in early stage HR+ breast cancer, regardless of luminal A or B status. Further research is warranted to evaluate the effect of palbociclib plus ET on nonluminal tumor subtypes as previous findings suggest that ET may be less beneficial in patients with nonluminal breast cancer compared with those with luminal cancers [39].

One of the most common sites for metastatic breast cancer is bone, with bone metastases present in approximately 80% of patients with metastatic breast cancer [40]. Supporting the present findings, a previous retrospective study found that various breast cancer subtypes are associated with different sites of metastases [41]. Compared with the HER2 and triple-negative subtypes, luminal A and luminal B subtypes were significantly associated with bone relapse, with 51% of patients with bone relapse having bone-only metastases [41]. Additionally, a significantly higher proportion of patients with luminal A subtype had bone-only metastases [41]. These preferential differences in metastatic sites may be explained by differentially expressed genes in patients with luminal subtype A or B versus other subtypes that dictate metastatic disease tropisms [41, 42].

In the present analysis of patients with bone-only disease, median PFS was prolonged with palbociclib plus ET compared with placebo plus ET in both PALOMA-2 and PALOMA-3. These findings are consistent with results from a previous analysis of PALOMA-2 and PALOMA-3 data that demonstrated improvement in PFS with palbociclib plus ET versus placebo plus ET in patients with bone-only disease (median PFS, PALOMA-2: NR vs 11.2 months; hazard ratio, 0.36; 95% CI 0.22‒0.59; *P* < 0.0001; data cutoff, February 26, 2016; PALOMA-3: 14.3 vs 9.2 months; hazard ratio, 0.63; 95% CI 0.38‒1.06; *P* < 0.05; data cutoff, October 23, 2015) [36].

## Conclusions

This analysis reinforces the benefit of palbociclib plus ET as a standard of care for patients with HR+/HER2− ABC, regardless of baseline TFI/DFI or intrinsic molecular subtype at the time of initial diagnosis or at the time of disease recurrence, including patients with bone-only disease, and suggests that according to both the published literature and currently available data, palbociclib plus ET should also be considered as an upfront treatment option in patients with more indolent disease. Additionally, *CDK4* gene expression was similar between patients with bone-only, nonvisceral (excluding bone-only), and visceral disease, but may be associated with resistance to single-agent letrozole. Further studies focusing on therapeutic strategies related to palbociclib sensitivity and development of resistance might clarify a more appropriate treatment sequence.

## Data Availability

Upon request, and subject to certain criteria, conditions and exceptions (see https://www.pfizer.com/science/clinical-trials/trial-data-and-results for more information), Pfizer will provide access to individual de-identified participant data from Pfizer sponsored global interventional clinical studies conducted for medicines, vaccines and medical devices (1) for indications that have been approved in the United States and/or European Union or (2) in programs that have been terminated (ie, development for all indications has been discontinued). Pfizer will also consider requests for the protocol, data dictionary, and statistical analysis plan. Data may be requested from Pfizer trials 24 months after study completion. The de-identified participant data will be made available to researchers whose proposals meet the research criteria and other conditions, and for which an exception does not apply, via a secure portal. To gain access, data requestors must enter into a data access agreement with Pfizer.

## Abbreviations

ABC: Advanced breast cancer
*CCND1*: Cyclin D1
*CCND3*: Cyclin D3
*CCNE1*: Cyclin E1
CDK: Cyclin-dependent kinase
*CDK4*: Cyclin-dependent kinase 4
*CDK6*: Cyclin-dependent kinase 6
CI: Confidence interval
CT: Computed tomography
DFI: Disease-free interval
*ESR1*: Estrogen receptor 1
ET: Endocrine therapy
FFPE: Formalin-fixed paraffin-embedded
HER2−: Human epidermal growth factor receptor 2–negative
HR: Hazard ratio
HR+: Hormone receptor–positive
IC: Informed consent
ITT: Intent-to-treat
MRI: Magnetic resonance imaging
LumA: Luminal A
LumB: Luminal B
NE: Not estimable
Non-Lum: Nonluminal
OS: Overall survival
PFS: Progression-free survival
STEPP: Subpopulation treatment effect pattern plot
TFI: Treatment-free interval
